# First in Asia: Ventricular tachycardia ablation in patients with mechanical aortic and mitral valves using right atrium to left ventricle approach

**DOI:** 10.1002/joa3.13186

**Published:** 2024-11-26

**Authors:** Bai Sitti Ameerah Asleah B. Tago, Chin‐Yu Lin, Ting‐Yung Chang

**Affiliations:** ^1^ Division of Cardiology, Department of Medicine, Heart Rhythm Center Taipei Veterans General Hospital Taipei Taiwan; ^2^ Department of Medicine National Yang Ming Chiao Tung University Taipei Taiwan

**Keywords:** catheter ablation, mechanical valve, heart failure, ventricular tachycardia

## Abstract

With the cases of mechanical valves, especially double mitral and aortic valves, ablation at the left ventricle is very challenging. This case report used equipments that are readily available in the Electrophysiology laboratory, which can make the access feasible.

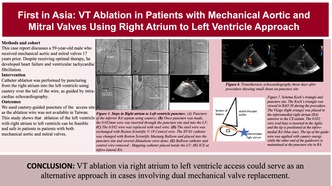

Mechanical aortic and mitral valves pose a challenge in left ventricle (LV) access. We present a case of successful catheter ablation for ventricular tachycardia (VT) via puncture of the inferior aspect of the right atrium (RA) without radiofrequency wires, which is not available in all countries in Southeast Asia. To the best of our knowledge, only Japan has RF wire in Southeast Asia and is not available in Taiwan. This marks the first use of this approach in Asia.

This is a case of 59‐year‐old male with aortic and mitral mechanical valves was admitted for syncope and episodes of ventricular tachycardia (VT)/ventricular fibrillation (VF) with implantable cardioverter‐defibrillator device (ICD) shock were recorded. The patient is nonhypertensive, nondiabetic, and has no previous history of myocardial infarction or underlying ischemic heart disease. The coronary arteries are patent, leading to an impression of nonischemic heart disease.

The procedure protocol consisted of Carto system, STSF ablation catheter, Decanav mapping catheter, Boston Scientific V‐18 Control wire 0.018 × 300 cm, Boston Scientific Mustang 10 mm × 40 mm × 135 mm, ST‐01 catheter, Agilis sheath, 0.032 mm wire, intracardiac echocardiography (ICE), Abbott, and TEE image. An Agilis sheath was inserted and placed in the right atrium (RA), then deflected toward the inferomedial RA. The tail of a 0.032 wire was inserted into the Agilis, with the tip positioned at the inferomedial RA, anterior to the coronary sinus (CS) ostium. With the intracardiac echocardiography (ICE) placed in the RA, we could visualize the CS, left ventricle (LV), and tricuspid valve (Figure 1F). The tip of the wire was positioned at the inferior septum of the RA at the desired puncture site. We applied 40 W from a standard electrocautery pen to the head of the wire, with each application lasting 3–5 s. An additional two cautery applications were performed until the wire successfully entered the LV. Episodes of VT were noted during the application of energy. The wire is then advanced into LV apex. A schema of Koch's triangle and puncture site is provided in Figure 2A. The Koch's triangle as viewed in RAO 30; and the Agilis sheath (light orange) is placed in the inferomedial RA anterior to the CS os. The 0.032 wire (red line) is inserted in the Agilis and the tip is positioned at the inferomedial RA (blue star).

**FIGURE 1:**
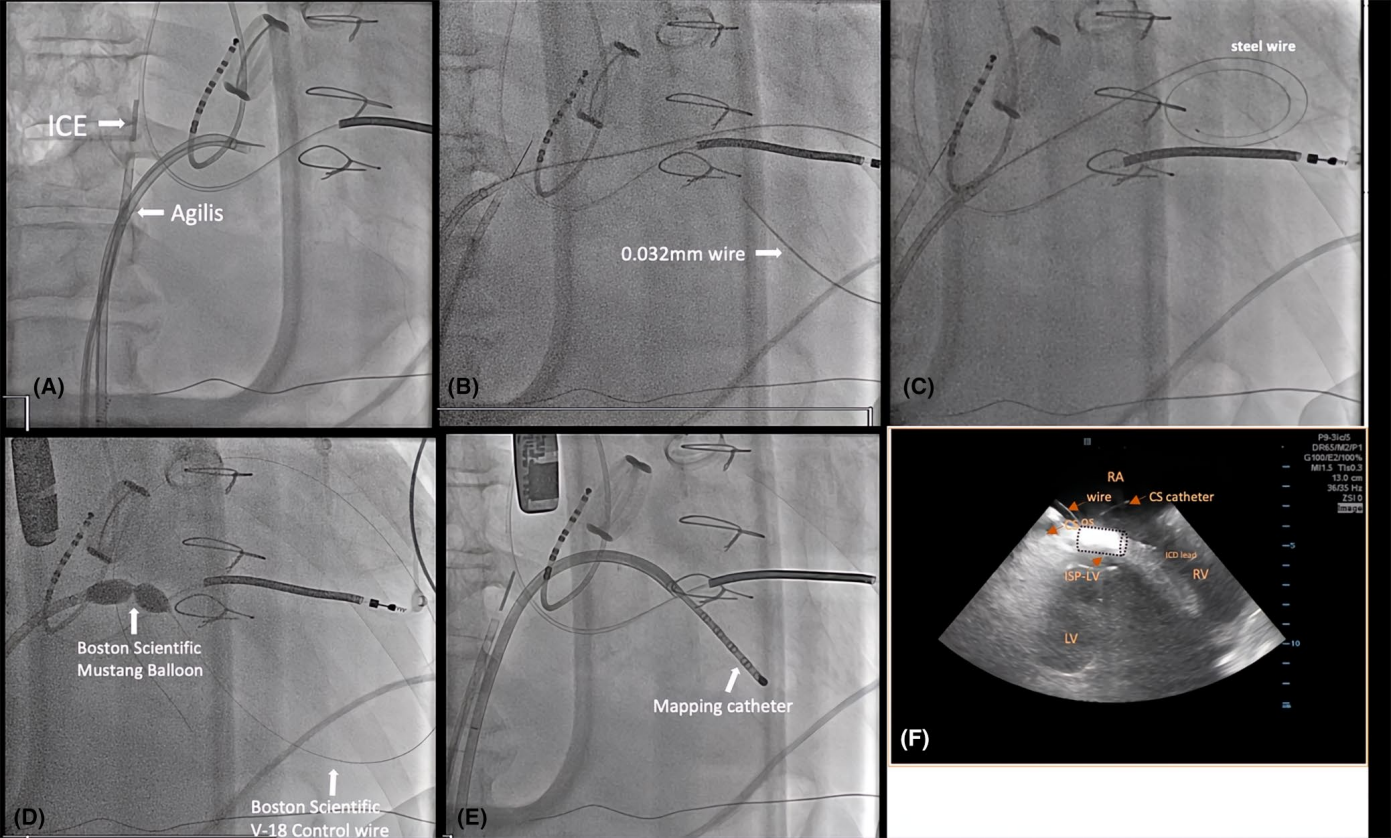
Steps in Right atrium to Left ventricle puncture. (A) Puncture of the inferior RA septum using cautery; (B) Once puncture was made, the 0.032 mm wire was inserted through the puncture site and into the LV; (C) The 0.032 wire was replaced with steel wire; (D) The steel wire was exchanged with Boston Scientific V‐18 Control wire. The ST‐01 catheter was changed with Boston Scientific Mustang Balloon and placed into the puncture site and several dilatations were done; (E) Balloon catheter and control wire removed; Mapping catheter placed inside the LV; (F) ICE at inferolateral RA. The 0.032 wire was positioned at inferior RA septum and visualized as it punctured into the LV. ICE, intracardiac echocardiography; LV, left ventricle: RA, right atrium.

**FIGURE 2:**
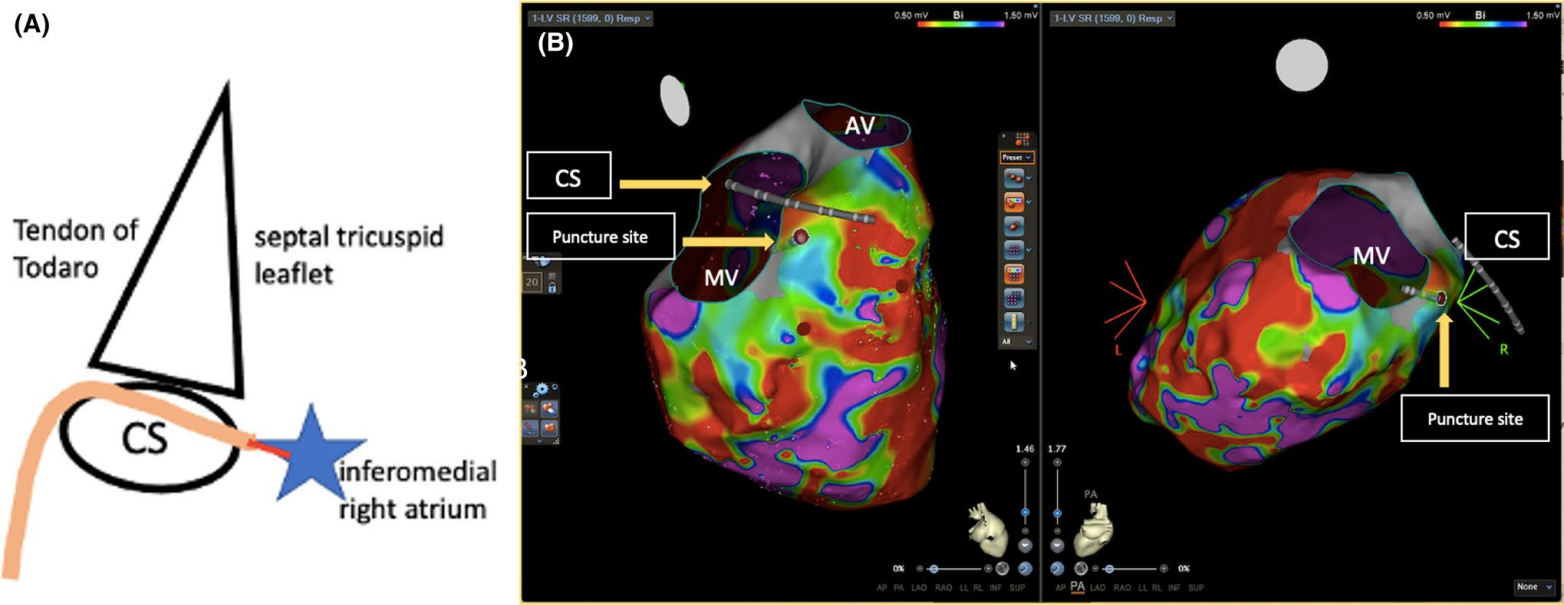
Schematic illustration and 3D image of Puncture site. (A) Schema Koch's triangle and puncture site. The Koch's triangle was viewed in RAO 30 during the procedure. The Agilis (light orange) was placed in the inferomedial right atrium (RA) anterior to the CS ostium. The 0.032 wire (red line) is inserted in the Agilis and the tip is positioned at the inferomedial RA (blue star). The tip of the guidewire was applied with cautery energy while the other end of the guidewire is maintained at the puncture site in RA. (B) 3D image of Right atrium to left ventricle puncture site. AV, aortic valve; CS, coronary sinus; MV, mitral valve.

During the puncture, VT was induced with right bundle branch block (RBBB) morphology and superior axis, which was terminated with cardioversion (Figure 3A). The puncture site can also be seen using 3D image as seen in Figure 2B. The 0.032 wire and ST‐01 catheter were advanced into the puncture site (Figure 1B). The 0.032 wire was replaced with steel wire (Figure 1C) and was exchanged for a Boston Scientific Control wire (Figure 1D). The ST‐01 was changed with a Boston Scientific Mustang balloon catheter (Figure 1D). Once puncture site was dilated, the Agilis was inserted into the LV with the dilator. Afterwards, the mapping catheter was positioned in the LV (Figure 1E), where late potentials were identified at the LV basal septum (Figure 3C). During the procedure, we performed pace mapping to match the VT morphology. Ablation was conducted at the VT exit, and we homogenized the area as seen in Figure 3B with local abnormal ventricular activities (LAVAs) during sinus rhythm or ventricular pacing according to our protocol.^1^

**FIGURE 3:**
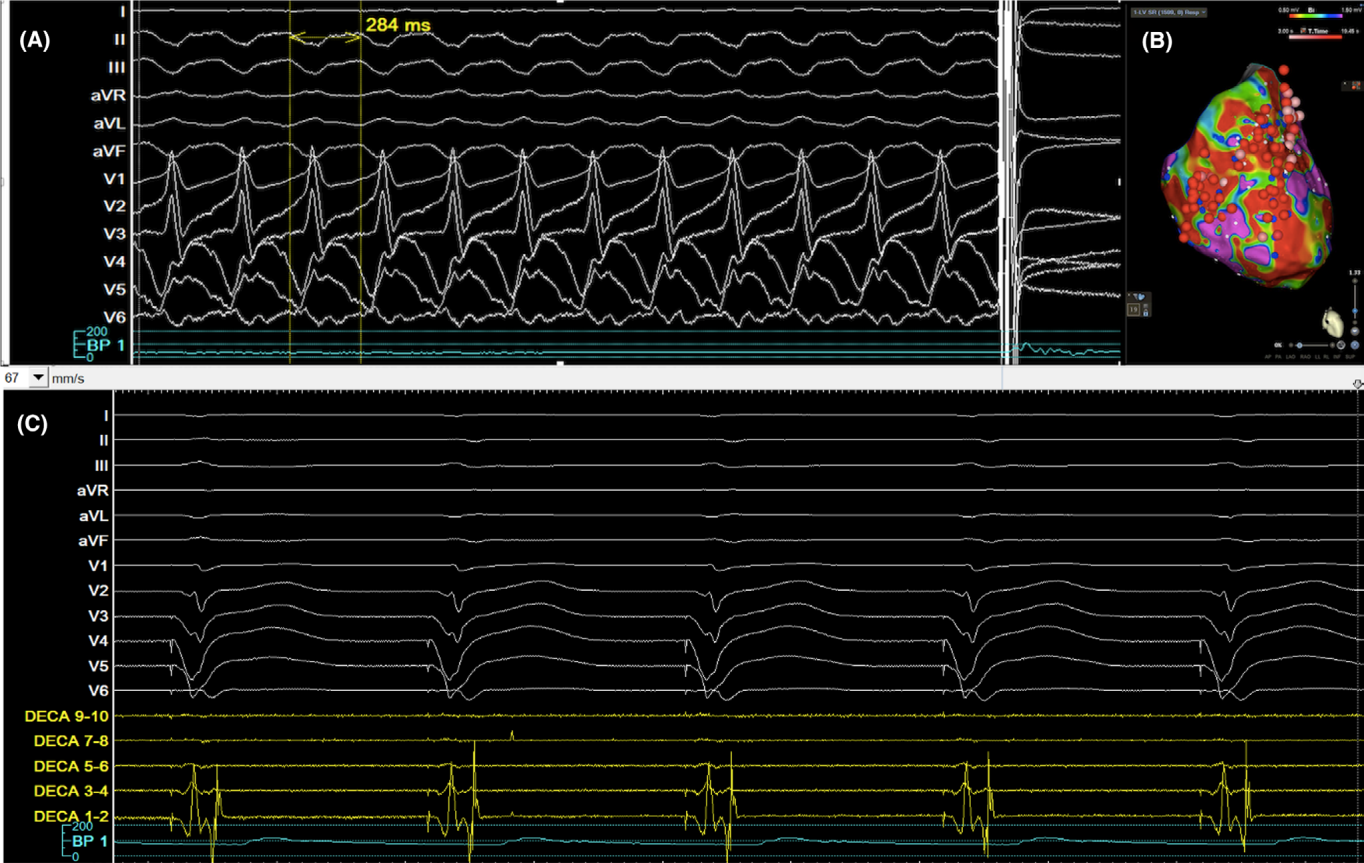
12 L EKG and intracardiac recordings during the procedure with ablation lesions on 3D. (A) 12 lead electrocardiogram of Ventricular Tachycardia induced during the right atrium to left ventricle puncture with tachycardia cycle length 284 ms, RBBB, superior axis and terminated with cardioversion. (B) Ablation lesions. Post ablation voltage map with ablation lesions on left ventricular septum. Red tags are ablation lesions. Color coding settings for bipolar voltage from 0.50 mV(red) to 1.50 mV(magenta). (C) Example of late potentials as seen on the decapolar mapping catheter (Deca 1–2 and 3–4) during pacing at the left ventricle (LV), particularly at basal septum in this tracing. Decapolar mapping catheter was positioned at LV basal septum.

On repeat echocardiography (Figure 4) 3 days during hospitalization and 1 month after discharge, a residual shunt was still noted with a Qp/Qs of 1.9 before and after the procedure but it seems that the Qp/Qs estimation via echocardiography might be an overestimate, which is a limitation to this case. Rheumatic heart disease could have contributed to the left to right shunt and is another limitation to this case. However, there was no sign of heart failure after the procedure until follow‐up.

**FIGURE 4:**
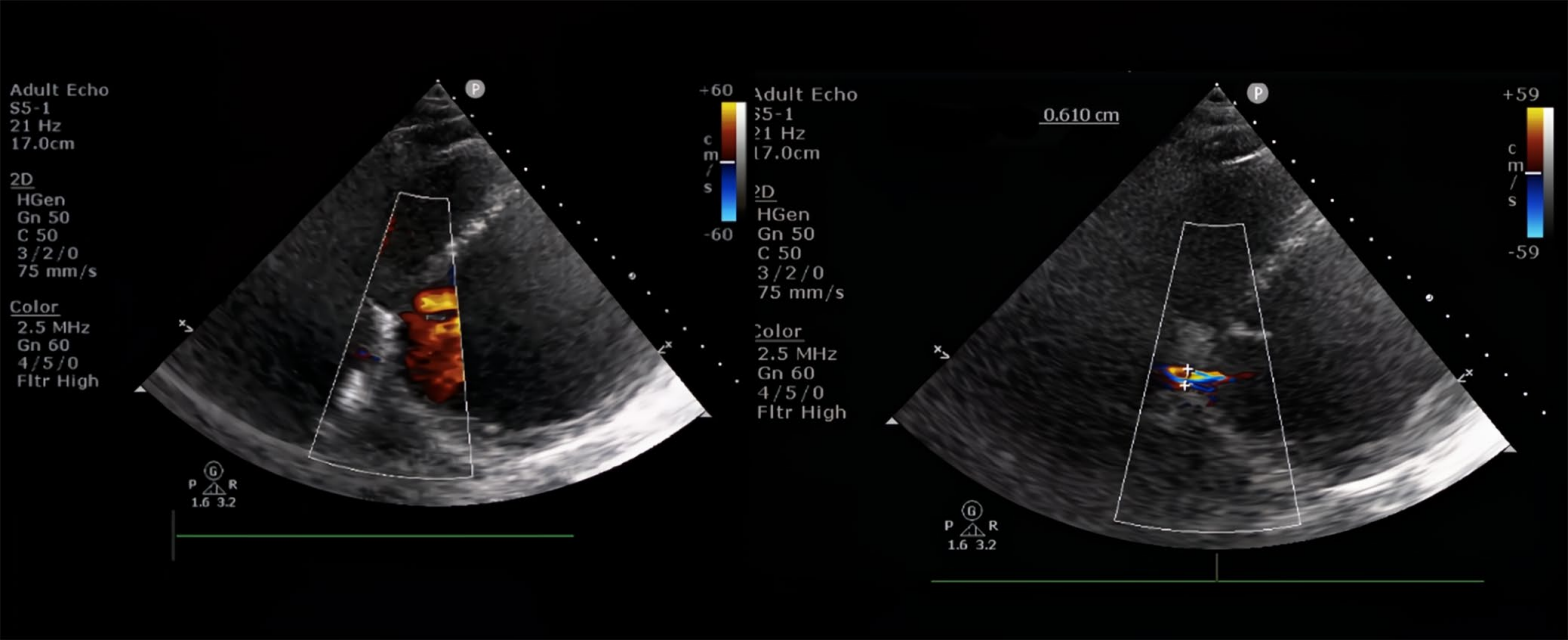
Transthoracic echocardiography 3 days after procedure showing shunt on puncture site.

Our patient has a similar approach to Dr. Santangeli's proposed technique.^2^ We modified the technique by using cautery‐guided puncture of the access site as the ablation wire was not available in Asia. No preprocedural imaging such as MRI or CT was done for this patient because of poor renal function and incompatible ICD lead. We also discussed the alternative approach (transapical puncture and epicardial approach) in a shared decision making method. The patient decided to undergo this approach first described in the article.

Double mitral and aortic mechanical valves present an access challenge when planning a ventricular tachycardia ablation as entry into the left ventricle is not possible via traditional approach. Other reported application of nonconventional techniques for ventricular tachycardia ablation include left ventricular assist device implantation prior to ablation, transapical LV procedure, or transventricular puncture. Catheter ablation of VT performed in patients with LVAD represents a treatment of last resort when ICD and drug therapy have failed.^3^ Transthoracic direct transapical access is associated with an unfavorable benefit–risk ratio in the published reports because of frequent and severe bleeding events. The surgical‐assisted transapical access shows a good risk–benefit ratio in the published reports and allows the immediate control of possible complications. Transvenous access can be through right atrium to left ventricle or interventricular transeptal access. In transvenous access routes, the limited accessibility of substrates near the puncture site and the risk of conduction disturbances, because of proximity to the conduction system, should be considered.^4^ His catheter can be placed to properly delineate the AVN. Puncture is done at the most inferior aspect of the inferior septal process of the LV. However, in our case, the puncture site at the level of the CS os at its base hence we assume it is far from AVN.

Recently, Santangeli et al. described a puncture technique from the right atrium into the inferior septal process of the LV. Santangeli et al. described the outcomes of their observational study which included four patients with recurrent VT associated with an LV substrate and mechanical valves in the aortic and mitral position using this puncture technique. On follow‐up, no patient had VT recurrence at a median follow‐up of 14 months.^2^ Although a small residual shunt was observed 3 days after the procedure, close follow‐up is necessary to monitor for any progression of the shunt. An immediate echocardiogram should be performed if sudden symptoms of decompensation occur. According to Santangeli, a transthoracic echocardiogram (TTE) should be repeated 24 h postprocedure and again at 4–8 weeks postprocedure.

A multicenter study was done by Siontis et al. regarding the feasibility, safety, and outcomes of right atrium to the left ventricle via puncture of the inferior atrio‐ventricular septum in patients with aortic and mitral mechanical valves. This involved 13 patients. In most patients (*n* = 10), LV substrate distribution was inferior/inferolateral. Postablation, complete noninducibility of VT was achieved in 12 (92%) patients. During median follow‐up of 8 months, 2 (15%) patients experienced VT recurrence.^5^

VT ablation via right atrium to left ventricle access could serve as an alternative approach in cases involving dual mechanical valve replacement. This is the first reported case in Asia using this approach.

## FUNDING INFORMATION

This work was supported by the Biosense Webster IIS (C2304900), Ministry of Science and Technology (MOST 110‐2314‐B‐A49A‐541‐MY3, MOST 111‐2314‐B‐075‐007‐MY3); and Taipei Veterans General Hospital (grant no. C19‐027). The funders did not involve the design of this study. C‐YL was the recipient of the funding award. The funders had no role in the study design, data collection and analysis, decision to publish, or manuscript preparation.

## CONFLICT OF INTEREST STATEMENT

The authors declare no conflict of interest.

## ETHICS STATEMENT

We obtained IRB approval for a retrospective study. In this study, we only reviewed the procedural details without any further intervention. Consequently, patient informed consent and ethical consent were waived. (Taipei Veterans General Hospital IRB number 2024–05‐009 BC).

## PATIENT CONSENT STATEMENT

Patient's informed consent has been waived by IRB approval.
